# Anterograde Tracing From the Göttingen Minipig Motor and Prefrontal Cortex Displays a Topographic Subthalamic and Striatal Axonal Termination Pattern Comparable to Previous Findings in Primates

**DOI:** 10.3389/fncir.2021.716145

**Published:** 2021-11-26

**Authors:** Johannes Bech Steinmüller, Carsten Reidies Bjarkam, Dariusz Orlowski, Jens Christian Hedemann Sørensen, Andreas Nørgaard Glud

**Affiliations:** ^1^CENSE, Department of Neurosurgery, Aarhus University Hospital, Aarhus, Denmark; ^2^Department of Clinical Medicine, Faculty of Health, Aarhus University, Aarhus, Denmark; ^3^Department of Neurosurgery, Aalborg University Hospital, Aalborg, Denmark

**Keywords:** basal ganglia, hyperdirect pathway, motor cortex, neuronal tracing, prefrontal cortex, striatum, subthalamic nucleus, *Sus scrofa*

## Abstract

**Background**: Deep brain stimulation (DBS) of the dorsal subthalamic nucleus (STN) is a validated neurosurgical treatment of Parkinson’s Disease (PD). To investigate the mechanism of action, including potential DBS induced neuroplasticity, we have previously used a minipig model of Parkinson’s Disease, although the basal ganglia circuitry was not elucidated in detail.

**Aim**: To describe the cortical projections from the primary motor cortex (M1) to the basal ganglia and confirm the presence of a cortico-striatal pathway and a hyperdirect pathway to the subthalamic nucleus, respectively, which is known to exist in primates.

**Materials and Methods**: Five female Göttingen minipigs were injected into the primary motor cortex (*n* = 4) and adjacent prefrontal cortex (*n* = 1) with the anterograde neuronal tracer, Biotinylated Dextran Amine (BDA). 4 weeks later the animals were sacrificed and the brains cryosectioned into 30 μm thick coronal sections for subsequent microscopic analysis.

**Results**: The hyperdirect axonal connections from the primary motor cortex were seen to terminate in the dorsolateral STN, whereas the axonal projections from the prefrontal cortex terminated medially in the STN. Furthermore, striatal tracing from the motor cortex was especially prominent in the dorsolateral putamen and less so in the dorsolateral caudate nucleus. The prefrontal efferents were concentrated mainly in the caudate nucleus and to a smaller degree in the juxtacapsular dorsal putamen, but they were also found in the nucleus accumbens and ventral prefrontal cortex.

**Discussion**: The organization of the Göttingen minipig basal ganglia circuitry is in accordance with previous descriptions in primates. The existence of a cortico-striatal and hyperdirect basal ganglia pathway in this non-primate, large animal model may accordingly permit further translational studies on STN-DBS induced neuroplasticity of major relevance for future DBS treatments.

## Introduction

The subthalamic nucleus (STN) is an important part of the basal ganglia circuitry, where it exerts and adjusts motor control by modulating the basal ganglia output of the internal globus pallidus (GPi) and substantia nigra pars reticularis (SNr; Alexander et al., 1990; Bonnevie and Zaghloul, 2019). Previously, the basal ganglia input from the striatum was perceived as diverging into two entities: the excitatory “direct pathway” promoting motor activity, and its counterpart, the inhibitory “indirect pathway”, involving the STN. Their opposite functions were mediated by different dopamine receptors. Later, this anatomical understanding of STN function was expanded by the introduction of the “hyperdirect pathway”, which was found to be somatotopically organized within the STN of primates (Nambu et al., 1996, 1997). In addition, it was hypothesized that the STN was not responsible for the generation of the movement itself, but instead permitted a selective action control by the inhibition of conflicting motor mechanisms (Mink, 1996). The basis for this was proposed to constitute a “center-surround-model” of sequential signal processing combining the hyperdirect, direct, and indirect pathways, which enabled inhibition of competing motor inputs and facilitation of selected motor actions (Nambu et al., 2002). Later, research has related the STN to cognitive and limbic functions as well (Krack et al., 2010; Hamani et al., 2017).

In movement disorders, including Parkinson’s disease (PD), the basal ganglia circuitry is compromised (Mallet et al., 2008; Gittis et al., 2011). Early research revealed that lesions in the basal ganglia ameliorated PD symptoms (Svennilson et al., 1960), which could subsequently be reproduced by high frequency deep brain stimulation (DBS) in the STN (Limousin et al., 1995, 1998). Today, DBS is increasingly being used as a neurosurgical treatment modality adjunct to the primary medical treatment of PD. Interestingly, this has not only benefitted patients but neuroscience research as well, since the placement of electrodes in the basal ganglia has yielded a unique opportunity to study its function in human patients (Knight et al., 2015; Miocinovic et al., 2018; Chen et al., 2020) as well as in translational research (Gradinaru et al., 2009; Anderson et al., 2018; Johnson et al., 2020). Through this research, increased insight into both PD pathophysiology and mechanisms underlying the effects of DBS has been achieved, and a possible role of antidromic activation of cortical areas through the hyperdirect pathway has been investigated although not yet fully understood (Johnson et al., 2020).

In recent years, the use of porcine models has increased due to economical and ethical advantages compared with primate studies (Goodman and Check, 2002; Lind et al., 2007; Sørensen et al., 2011). Accordingly, the neuroanatomy of the Göttingen minipig is gradually being characterized (Larsen et al., 2004; Meidahl et al., 2016; Bjarkam et al., 2017a; Bech et al., 2018, 2020), which has facilitated its use in translational studies of PD (Glud et al., 2010, 2011; Lillethorup et al., 2018). Also, the small size of the minipig permits the use of clinical scanners, even in longitudinal studies, as well as human surgical equipment including DBS (Bjarkam et al., 2008; Ettrup et al., 2012), which has been used to alleviate symptoms in translational models of PD (Christensen et al., 2018).

To further strengthen the porcine translational studies of PD using DBS, this study aims to describe the cortico-striatal and hyperdirect pathways to the basal ganglia by the use of anterograde neuronal tracing. The existence and characterization of such will elucidate an important part of the basal ganglia circuitry and hence yield an anatomical rationale for investigating the underlying mechanisms of DBS including cortical neuroplastic changes in a non-primate, large animal model.

## Materials and Methods

In this study, we further analyzed the histological sections from the brains of five minipigs obtained in a previous study (Bech et al., 2018). See specifics below.

### Animals

Five female Göttingen minipigs (Ellegaard Göttingen Minipigs, Dalmose, DK), numbered JBG1–5, aged between 11 and 15 months, and weighing 22.6–28 kg were used in this study as approved by the Danish National Council of Animal Research Ethics (protocol number 2015-15-0201-00965).

### Neuronal Tracing

A high molecular weight biotinylated dextran amine (BDA, 10 kDa; NeuroTrace*^TM^* BDA-10.000 Neuronal Tracer Kit, ThermoFischer, Waltham, MA, USA) was used as an anterograde neuronal tracer. The tracer was freshly mixed in 0.01 M phosphate buffered saline (pH 7.4) yielding a 10% BDA solution prior to each injection for optimal stability.

### Stereotaxic Surgery

Animals were initially sedated with an intramuscular injection of midazolam (6 ml, 5 mg/ml, Hameln^®^) and ketamine (4 ml 25 mg/ml, Pfizer^®^). Intravenous access was obtained through cannulation of an ear vein which permitted a subsequent intravenous dose of sedation. The animals were then intubated as previously described (Ettrup et al., 2011) and placed in an MRI-compatible head frame, where they were fixated with zygomatic screws (Bjarkam et al., 2004). Anesthesia was then continued with 2% sevoflurane. Animals received buprenorphine analgesics (Temgesic^®^) and antibiotics (Cefuroxim “Fresenius Kabi” 1.5 g), whereafter marcaine infiltrative analgesics were injected subcutaneously in the skull midline and at the site for the zygomatic screws. A midline incision was made, and the skull exposed for fiducial marker placement in a skull burr hole (Glud et al., 2017). Animals were then MRI scanned (Siemens 3T TIM Trio) to obtain anatomical sequences for fiducial marker visualization (Flash 3D T1-weighted sequence, slice thickness 1 mm, voxel size 1 × 1 × 1 mm^3^, 176 slices, *FOV* = 256 mm, *TR* = 2420 ms, *TE* = 3.7 ms, 2 averages, *TI* = 960 ms, and flip angle = 9 degrees). Stereotaxic coordinates were defined in the center of the primary motor cortex (M1) on MRI software (Bjarkam et al., 2009). The stereotaxic system was attached to the head frame and a craniotomy was made over the estimated injection site. The dura was opened with a dura knife and the cortex exposed. The stereotaxic entry point at the cortex was evaluated against previously defined macroscopic surface anatomy (Bjarkam et al., 2017a) to visually identify the M1/dorsal prefrontal cortex (dPFC) gyrus, whereafter injections could be placed correctly during open surgery. Using a Hamilton microsyringe, nine microinjections, each of 0.5 μl yielding a total dose of 4.5 μl, were slowly pressure injected (0.1 μl/min) using a micromanipulator (three sites at the target area, 1 mm apart—injected at a depth of 1, 2 and 3 mm below the cortical surface). After each injection, the syringe was left for 5 min before retracting to prevent reflux of tracer leading to unintended uptake of tracer from adjacent cortical areas. The dura and skin were then closed and sutured.

### Tissue Handling

26–31 days after injection of BDA animals were euthanized by a pentobarbital overdose with prior sedation. Followingly, the brain tissue was fixated by transcardial perfusion of 5 L phosphate buffered formaldehyde (PFA, 4% solution, pH 7.4) as previously described (Ettrup et al., 2011). The brains were removed (Bjarkam et al., 2017b) and the injected cortical area with a compromised dural opening was identified and labeled with tissue staining (India Ink^®^). Brains were then placed in a 4% PFA solution. Brains were transferred to a PBS solution followed by embedding in a supportive alginate polymer before being cut into 1.5–2 cm thick coronal blocks (Bjarkam et al., 2001). The blocks were submerged into 30% sucrose for approximately 8–14 days, until they were no longer floating. Tissue blocks were then frozen in liquid isopentane cooled by dry ice and cut in 30 μm sections on a cryostat in series where every tenth section was kept for analysis.

### Histology

Sections were placed in phosphate buffer and quenched with 10% H_2_O_2_ and 3% methanol phosphate buffer solution to block endogenous peroxidase. They were then incubated in bovine serum albumin before being transferred to an avidin-biotin-peroxidase complex solution (VECTASTAIN^®^ Elite ABC kit, Vector Laboratories, Burlingame, CA, USA). The BDA was visualized using DAB solution (Kem-En-Tec Nordic A/S, Taastrup, Denmark). Sections were mounted on glass slides, counterstained with toluidine blue, and coverslipped. Finally, sections underwent microscopic analysis, using a Leica DM5000B microscope. We divided the STN into a respective anterior, central and posterior segment according to section topography as this anteroposterior classification has previously been made for the STN in other studies (Haynes and Haber, 2013). Likewise, the striatum was analyzed in three similar segments in the antero-posterior axis, an anterior, central, and posterior, respectively.

## Results

The tracing data is summarized in Table 1 for an overview. The respective anatomical regions are described in detail below. For an overview of cortical injection sites, see Supplementary Figure 1.

**Table 1:** Schematic summary of the neuronal tracing found across different regions or nuclei.

			JBG-1	JBG-2	JBG-3	JBG-4	JBG-5
		Injection	M1	dPFC	M1	M1	M1
		Thalamic nucleus^†^	VA/VL	MD	VA/VL	VA/VL	VA/VL
	**Nucleus/Region**	**Part**					
*Striatum*	**Caudate**	Anterior	+	+++	++	+++	++
		Central	+	+++	+++	+++	+++
		Posterior	-	++	+	++	-
	**Putamen**	Anterior	+++	+++	+++	+++	+++
		Central	+++	++	+++	+++	+++
		Posterior	-	+	+	+++	-
	**STN**	Anterior	++*	+^§^	++*	+*	-
		Central	+++*	+++^§^	+++*	+*	+*
		Posterior	++*	++^§^	+++*	++*	+*
	**ZI**	-	+	+	++	+	+
	**SNc**	-	+	++	+++	+	+
	**NAcc**	-	-	+++	++^‡^	++^‡^	+^‡^
	**vPFC**	-	-	++	-	-	-
	**dPFC (contralateral)**	-	+	+++	++	++	+
	**M1 (contralateral)**	-	++	+++	++	+++	++
	**Amygdala**	-	-	++	-	-	-

**Figure 1:**
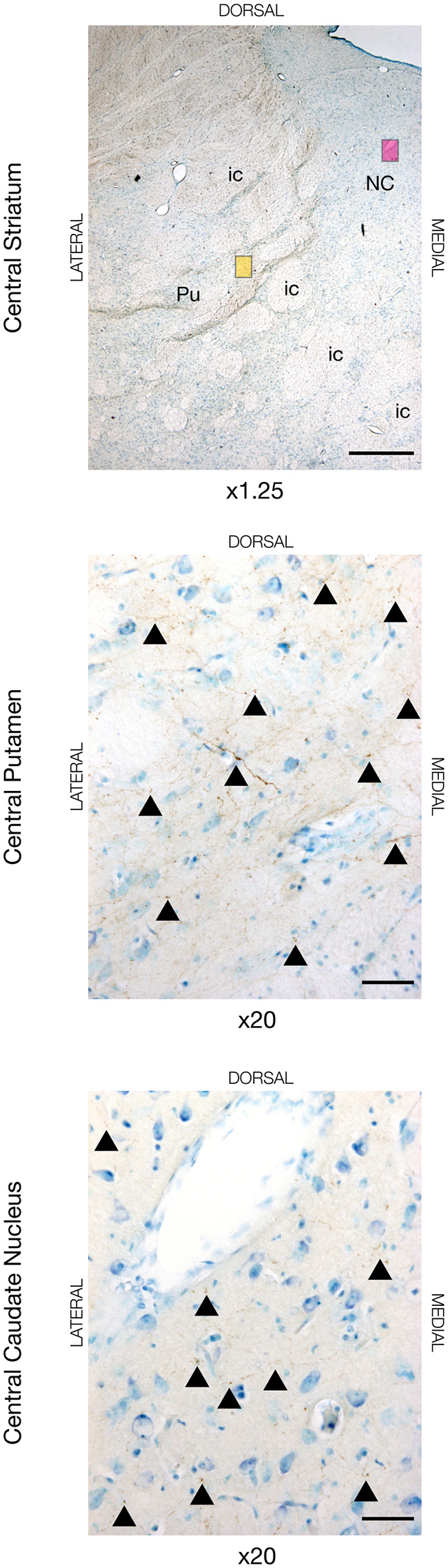
Neuronal tracing with Biotinylated Dextran Amine (BDA) in the dorsal striatum in JBG-3. The upper image is for overview displaying the central segment of the striatum with the putamen (Pu), caudate nucleus (NC), and intersecting internal capsule (ic). The yellow miniature window is showing the area in the dorsal putamen seen in x20 magnification on the middle image. The magenta miniature window is showing the area in the dorsal caudate nucleus seen in x20 magnification on the bottom image. Note the golden-brown traced fibers revealing numerous axons and terminations marked with arrowheads. Counterstaining with toluidine blue. Scale bars = 1 mm (x1.25) and 50 μm (x20).

Tracing is arbitrarily organized accordingly to amount of found fibers: (+++) many fibers/diffuse terminations (e.g., as seen in Figure 1), (++) several fibers (e.g., as seen in zona incerta in Figure 4), (+) few fibers (e.g., as seen in septum in Supplementary Figure 4), and (-) no fibers. STN, subthalamic nucleus; ZI, zona incerta; SNc, substantia nigra pars compacta; NAcc, nucleus accumbens; vPFC, ventral prefrontal cortex; dPFC, dorsal prefrontal cortex; M1, primary motor cortex. ^†^Data from previous study with permission from Bech et al. (2018). *Predominantly lateral STN. ^§^Predominantly medial STN. ^‡^Fibers in the transition zone between NAcc and putamen.

### Cortico-Cortical Tracing

In four animals (JBG1,3-5) the cortical injection was histologically evaluated to be within the M1 by combining section topography, cytoarchitecture, and the presence of large pyramidal cells of Betz as previously described (Bech et al., 2018). The tracing here was found to label both association fibers to neighboring cortical areas as well as commissural fibers to the contralateral motor cortex and dorsal prefrontal cortex (dPFC). The remaining animal (JBG2) was found to be more anteriorly injected in the dPFC, where a different cytoarchitecture and no pyramidal cells of Betz were present. Tracing from the dPFC was, likewise, found in adjacent cortical areas as associating fibers, but formed more extensive commissural connections to both the contralateral dPFC as well as M1.

### The Cortico-Striatal Pathway

To delineate the cortico-striatal pathway that constitutes the initial common part of the direct/indirect pathways (Nambu et al., 2002), we evaluated the cortical projections to the putamen and caudate nucleus. The motor cortical connections to the putamen were predominantly found in the dorsal and lateral part, where the majority of fibers were found in the anterior and central segment of the striatum with gradually lesser tracing found in the posterior direction, see Figure 1. A substantial termination of motor fibers was found deemed by closely packed fiber networks and boutons. Likewise, most of projecting motor fibers to the caudate nucleus was found in the most dorsal part of the caudate nucleus and in close relation to the laterally traversing white matter of the internal capsule. Fibers formed numerous axonal termination networks and boutons in this area, but there seemed to be most tracing in the central segment of the caudate nucleus. Additionally, in the two animals most anteriorly injected in the M1 (JBG-3+5, see also Bech et al., 2018), tracing was also found more central in the caudate nucleus. The amount of tracing in the putamen generally exceeded that found in the caudate nucleus. Interestingly, the animal injected in the dPFC displayed different connectivity with extensive fiber terminations in the mediodorsal part of the anterior and central caudate nucleus, and, to a lesser extent, in the posterior caudate nucleus. In this animal, fewer fibers were also found in the putamen, where they were more medially located when compared with the motor input.

### The Cortico-Subthalamic Hyperdirect Pathway

**Figure 2:**
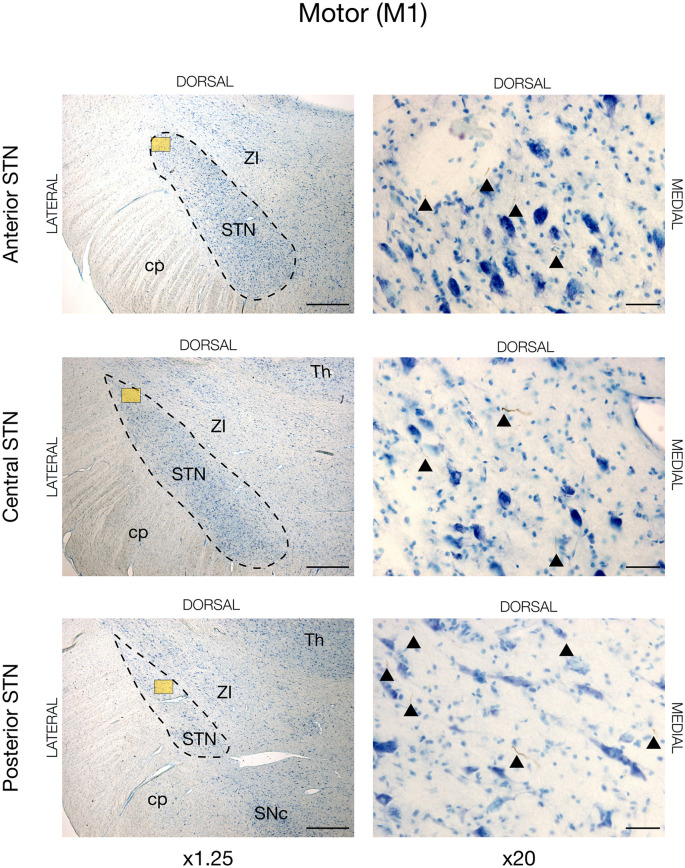
Motor projections from the primary motor cortex (M1) injected with the neuronal tracer, BDA, as seen in the subthalamic nucleus, STN (dashed lines), of JBG-1. The three rows display the anterior, central, and posterior segment, respectively, which is depicted in x1.25 magnification for overview in the left column. Colored miniature windows are the areas seen in detailed x20 magnification in the right column. Arrowheads mark the golden-brown traversing fibers of varying diameter. ZI, zona incerta; cp, cerebral peduncle; Th, thalamus; SNc, substantia nigra pars compacta. Counterstaining with toluidine blue. Scale bars = 1 mm (x1.25) and 50 μm (x20).

In all animals, there was visible tracing in the STN, but the BDA labeling was remarkably weaker in the STN when compared to the striatum and cortex. This weaker STN BDA labeling was in contrast to the strong BDA labeled fibers in the adjacent crus cerebri, which had a similar distance to the injection site. Motor cortical connections were predominantly found in the lateral and dorsal STN with the majority residing within the center and posterior segment, and, to a lesser degree, the anterior segment, see Figures 2, 5 (and Supplementary Figure 2). The terminations were not as abundant as in the striatum and some thicker axonal fibers were seen here. Contrarily, the prefrontal connections were almost exclusively projecting to the medial half of the STN (and Supplementary Figure 3).

**Figure 3:**
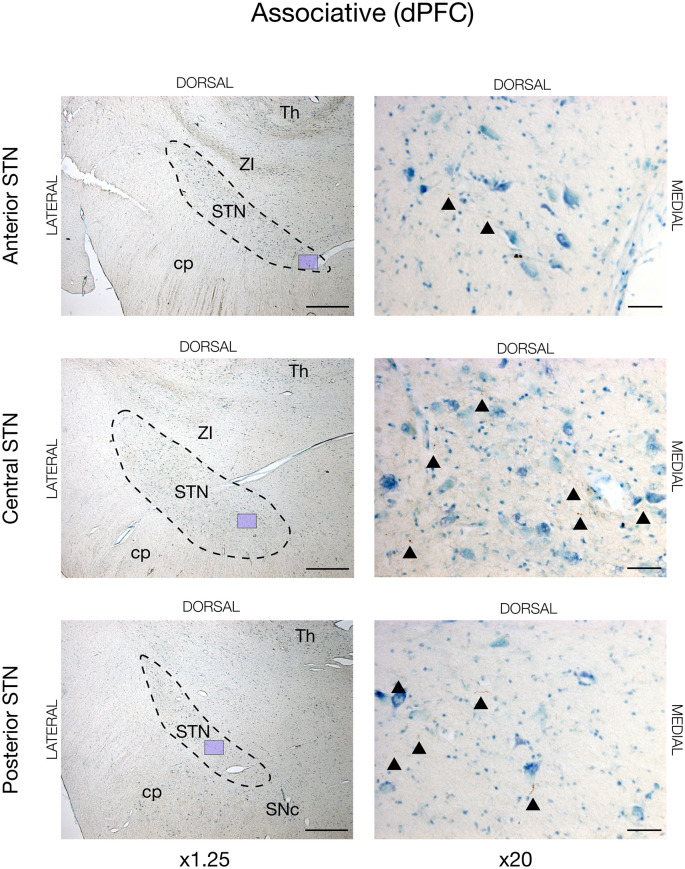
Associative projections from the dorsal prefrontal cortex (dPFC) injected with the neuronal tracer, BDA, as seen in the subthalamic nucleus, STN (dashed lines), of JBG-2. The three rows display the anterior, central, and posterior segment, respectively, which is depicted in x1.25 magnification for overview in the left column. Colored miniature windows are showing the areas seen in detailed x20 magnification in the right column. Arrowheads mark the golden-brown traversing fibers of varying diameter. ZI, zona incerta; cp, cerebral peduncle; Th, thalamus; SNc, substantia nigra pars compacta. Counterstaining with toluidine blue. Scale bars = 1 mm (x1.25) and 50 μm (x20).

**Figure 4:**
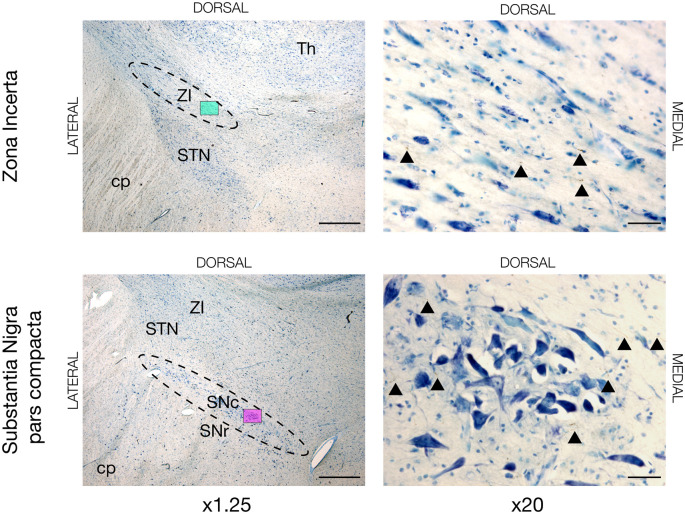
Neuronal tracing with BDA in the zona incerta (ZI) and substantia nigra pars compacta (SNc) of JBG-3. The left overview column is shown in x1.25 magnification marked with a colored miniature window, which is then shown in x20 magnification in the right column. BDA labeled axons are golden-brown and marked with arrowheads. STN, subthalamic nucleus; cp, cerebral peduncle; Th, thalamus; SNr, substantia nigra pars reticulata. Counterstaining with toluidine blue. Scale bars = 1mm (x1.25) and 50 μm (x20).

**Figure 5:**
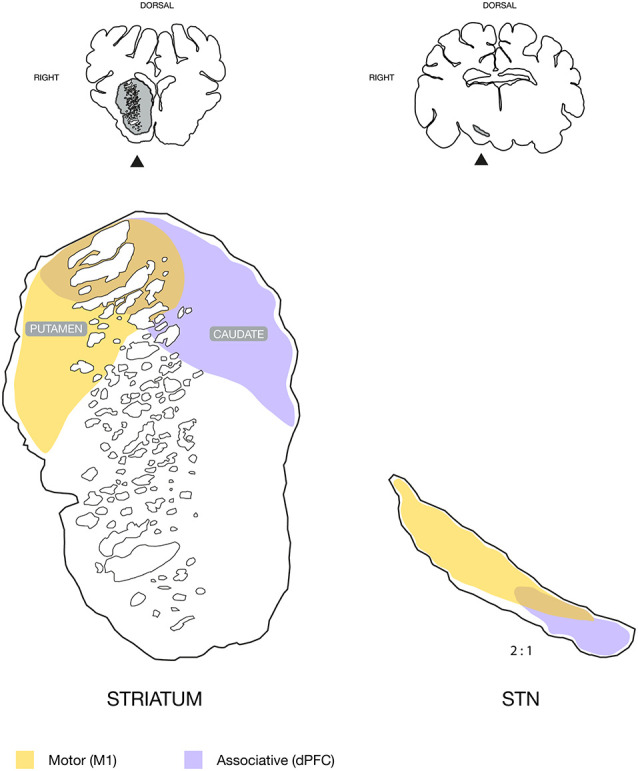
Schematic illustration of the topography found in the striatum and subthalamic nucleus (STN), with neuronal tracing. Motor input domains were labeled by the approximated combination of neuronal tracing patterns from animals injected in the primary motor cortex, M1. The associative domain represents the animal injected in the dorsal prefrontal cortex, dPFC. Coronal sections are illustrated above for overview with the striatum and STN marked in gray and with an arrow. Note that the STN is displayed in twice its size relative to the striatum for illustrative purposes.

### Other Regions With Motor Input

Aside from the cortical commissural and association connections, we discovered motor cortex projection fibers in other regions. For instance, tracing was seen in the zona incerta (ZI) and substantia nigra pars compacta (SNc) in all four animals, see Figure 4. In some animals (JBG3+4+5) traced termination axons were seen in the transition zone between the dorsolateral nucleus accumbens (NAcc) and most ventromedial putamen, see Supplementary Figure 4. Thalamic connectivity and the corticospinal tract were previously described (Bech et al., 2018).

### Other Prefrontal Connectivity

In accordance with the non-motor function of the dPFC, traced fibers were evident in the ventral prefrontal cortex (vPFC), see Supplementary Figure 5. As for the motor projections, there were also terminating axons in the NAcc, where fibers were somewhat more numerous and ventro-medially located than the motor input. Interestingly, traced fibers were seen in the basolateral nucleus of the amygdala and a few traced fibers were also seen near the septum, see Supplementary Figure 4.

## Discussion

Our main findings shed new light on the cortical projections to the basal ganglia of the Göttingen minipig. The primary motor cortical area was found to project predominantly to the dorsolateral putamen and adjacent caudate nucleus, which together with the dorsolateral STN formed a motor domain. Contrarily, an associative domain of the dPFC was found mainly to consist of the mediodorsal caudate nucleus, as well as the adjacent juxtacapsular dorsal putamen, and the medial STN as illustrated in Figure 5.

### Cortico-Striatal Pathway Similar to Primates

Our delineation of cortical motor efferents to the dorsolateral striatum is in accordance with the classically described segregation of the striatum into a dorsal sensory-motor part and ventral limbic part, and, furthermore supports the more recent oblique dorsolateral-ventromedial segregation (Voorn et al., 2004). In the antero-posterior axis, most efferents were found in the anterior and central part of the striatum with gradually diminishing tracing in the posterior direction. The tracing was predominantly found in the putamen, and to a lesser degree in the juxtacapsular part of the dorsal caudate. This finding is consistent with the small dorsolateral juxtacapsular caudate having been described not to be a part of the associative striatum that otherwise includes a large part of the remaining caudate nucleus (Parent and Hazrati, 1995). The traced fibers to the striatum were smaller and seen with numerous boutons and termination networks, which were clearly differentiable from the larger projecting axons within the perforating internal capsule white matter. In primates, motor efferents have also been found projecting to the putamen (Künzle, 1975; Jones et al., 1977; Liles and Updyke, 1985), where a somatotopical topography is present (Künzle, 1975; Parent, 1990). The termination pattern of our tracing data resembles that of the hindlimb in primates (Künzle, 1975), but as our study did not use electrophysiological determination of cortical representation, our data do not permit us to evaluate a possible somatotopic distribution. Interestingly, the fibers from the dPFC had entirely different connectivity, where abundant termination networks were found across a large mediodorsal part of the caudate and to a lesser degree in the juxtacapsular putamen. Together with previously found cortico-thalamic projections (Bech et al., 2018) from the dPFC to the mediodorsal thalamus, which were contrary to motor domains in the ventro-anterior and ventro-lateral thalamus (Bech et al., 2018), this clearly segregates the two cortical entities of the dPFC and M1. The basal ganglia have been proposed to constitute a “tripartite model” of functional subdivision into the respective motor, associative, and limbic circuitry (Krack et al., 2010; Hamani et al., 2017). Our data thus support this topographic model as the different connectivity found in the dPFC and M1 areas align with the associative and motor circuits, respectively, see Figure 5.

### Ventral Striatal Connectivity

Besides the abovementioned striatal connections, we also found tracing in the ventral striatum. A previous study of the NAcc in the Göttingen minipig has described the anatomic structure and retrograde connections of this ventral striatal area (Meidahl et al., 2016). Here, the NAcc was found to receive input from the medial aspects of the PFC, however, the dorsal PFC was not described to have any retrograde tracing. Conversely, our anterograde tracing from the dPFC displayed significant traced fibers in the NAcc. More uncertain is the motor cortical tracing found near the ventrolateral parts of the ventral striatum, where a sharp distinction between the putamen and dorsolateral NAcc does not exist (Meidahl et al., 2016). Thus, it is possible that the small amount of tracing from the M1 found in this area actually is part of the abutting ventrolateral putamen (Schmidt, 2015), where the face representation has been described to be in rodents (Voorn et al., 2004). This distinction is further complicated by the different anatomy of the porcine lentiform nucleus in comparison with that of the primates, and earlier studies have proposed the entopeduncular nucleus as a globus pallidus analogue (Min et al., 2012).

### Hyperdirect Pathway to STN

In all animals, we found cortical tracing in the STN. Generally, the tracing was less apparent than in the striatum, and, mostly, we found isolated traversing fibers. With few exceptions, diffuse termination networks were not visible, as was abundantly seen in the striatum, although axons of different diameters were present. The primary motor cortical efferents were mainly projecting to the dorsal and lateral part of the STN. This bears resemblance with findings in primates, where a motor domain has also been placed in this STN fraction (Monakow et al., 1978; Nambu et al., 1996). In primates, a somatotopic arrangement actually exists where the most lateral facial area is followed medially by the upper limb and then the lower limb area (Monakow et al., 1978; Nambu et al., 1996). Such an arrangement was not evident from our study as no clear segregation of the tracing patterns was found that could be correlated to specific cortical areas. This may have been due to our relatively widespread cortical tracer injections, which may have covered more than a specific cortical area. Also, the lack of clear terminal fields distinguishable from traversing fibers complicated a clear distinction. Interestingly, the dPFC tracing was more medially located in the STN than the motor projections, and dPFC projections were found in the center of the STN traversing gradually medial to reside in the medial fraction. This is in accordance with the dPFC projecting pattern described in a primate tracing study (Haynes and Haber, 2013), which hence suggests the possible existence of a porcine cognitive or associative domain in the STN, see Figure 5. Other cortical regions projecting to the medial STN in primates are the premotor cortex (PMC) and supplementary motor cortex (SMC; Nambu et al., 1996, 1997). A clearly defined PMC and SMC is, to the authors’ best knowledge, currently not well-described in pigs, but a study has proposed its location posteriorly to the dPFC (Jelsing et al., 2006). It is a possibility that some medial STN tracing from the prefrontally injected animal may originate from either a PMC or even an SMC area. The remaining tracing from the dPFC, however, displays connectivity that does not bear the resemblance of a motor, premotor or supplementary motor cortical area (Künzle, 1975; Nambu et al., 1996, 1997), e.g., with its intensive staining of the caudate nucleus.

### Cortical Connectivity

Both the prefrontal and motor cortical areas were found to have associative and commissural fiber networks. The dPFC displayed the highest degree of contralateral commissural connectivity, but also motor areas were interhemispherically connected. Reciprocal connectivity was also found between the dPFC and M1. As a possible premotor area could be interposed between the M1 and dPFC (Jelsing et al., 2006), this is of particular interest since novel research is beginning to decipher the neuronal mechanisms in cortical areas involved in motor planning (Li et al., 2015, 2016; Svoboda and Li, 2018), which involves the premotor area. Still, further studies are needed to perform similar experiments in pigs, since rodents are yet superiorly characterized.

### Methodological Considerations

Neuronal tracing is a classic and well-established method to study neuronal connectivity. Among other tracers, BDA is a successor to previous degenerative silver staining techniques, and it has proven to be a highly sensitive anterograde tracer able to yield an almost Golgi-like detail of neuronal fibers (Brandt and Apkarian, 1992; Reiner et al., 2000; Lazarov, 2013). Although more advanced techniques have emerged (Wouterlood et al., 2014), the BDA labeling in our study still proved to be clear and distinguishable from the background staining by the presence of BDA labeled axon terminal boutons. A methodological pitfall is, however, the possibility of drawing false negative conclusions based on the apparent absence of neuronal tracing, which is indeed present. This is especially the case for small diameter axonal fibers in areas with sparse connectivity, where these can easily be overlooked during the microscopic analysis. While this may not be crucial in the interpretation of the connectivity of the injected anatomical structure, it is still an important consideration. We have addressed issues of unspecific tracing, e.g., unintended adjacent tracer uptake, by hindering reflux of tracing after the cortical injections. Even so, theoretically, some tracer could have spread to the dPFC from the most anterior M1 injections and* vice versa*. Our results were, however, not indicative of this. The amount of prefrontal tracing data suffers from the single injected animal, but we chose to include these data as they serve the purpose of delimiting the primary motor cortex from the anteriorly positioned dPFC. Moreover, the dPFC connectivity was found to resemble what has been described in primates (Haynes and Haber, 2013).

### Conclusions and Perspectives

In our study, we have, for the first time, verified the presence of both a cortico-striatal pathway and a hyperdirect pathway from the cortical areas to the STN in the minipig. The traced fibers from the dPFC and motor cortex found in our data display a similar distribution as the respective associative and motor domains that have been found in primates. This finding points towards a comparable functional topography as described in the “tripartite model” of the basal ganglia function, which may therefore also be present in the minipig. Moreover, the common cortico-striatal part of the direct and indirect basal ganglia pathways as well as the hyperdirect pathway of the earlier proposed center-surround model have been outlined in our tracing data. This interesting model of action and motor control, which have subsequently been supported by a rodent study of action cancellation (Schmidt et al., 2013), may hence be reproducible in minipigs if the remaining parts of the direct and indirect pathways through the globus pallidus, or the entopeduncular nucleus, can also be outlined. Future studies are needed to determine this. Still, our findings suggest a great potential for the use of minipigs in translational studies of basal ganglia function and disorders as an alternative to primate studies. From a more clinical perspective, the presence of a hyperdirect connection between the STN and cortical areas constitute an anatomical rationale to investigate the cortical effects of STN DBS including the possible induction of neuroplastic changes. Such translational models may hence aid in revealing the yet obscure underlying mechanisms of DBS treatment for the future benefit of an increasing number of patients treated with this neurosurgical modality.

## Data Availability Statement

The datasets presented in this article are not readily available because the data is derived from not publicly available histology sections. Requests to access the datasets should be directed to jb@clin.au.dk.

## Ethics Statement

The animal study was reviewed and approved by the Danish National Council of Animal Research Ethics (protocol number 2015-15-0201-00965).

## Author Contributions

AG, CB, JSø, and JSt conceived and designed the study. JSt, AG, JSø, and CB performed the surgery and stereotaxic tracer injection. JSt, CB, and DO performed the data analysis. JSt and DO made the figures. All authors contributed to the writing of the manuscript. All authors contributed to the article and approved the submitted version.

## Funding

We are grateful for the funding received from the Novo Nordisk Foundation (Grant no. NNF15OC00015680), the Jascha Foundation (Grant no. 5559), “Fonden for Neurologisk Forskning”, and “Simon Fougner Hartmanns Familiefond”, which permitted us to do the study.

## Supplementary Materials

The Supplementary Material for this article can be found online at: https://www.frontiersin.org/articles/10.3389/fncir.2021.716145/full#supplementary-material.

Supplementary Figure 1 Overview of the cortical injection sites in animals JBG1-5. Note the golden-brown BDA labeling of the cortical areas of M1 and dPFC, respectively, marked with arrowheads. Counterstaining with toluidine blue. Scale bars = 5 mm.

Supplementary Figure 2 Neuronal tracing with BDA in JBG-1. The figure depicts an absence of motor projections from the primary motor cortex (M1) to areas of the medial subthalamic nucleus, STN (dashed lines). The three rows display the anterior, central, and posterior segment, respectively, which is depicted in x1.25 magnification for overview in the left column. Colored miniature windows are the areas seen in detailed x20 magnification in the right column, where no traced fibers are seen in the medial STN. Only sparse motor projections where found elsewhere in the medial STN. ZI, zona incerta; cp, cerebral peduncle; Th, thalamus; SNc, substantia nigra pars compacta. Counterstaining with toluidine blue. Scale bars = 1 mm (x1.25) and 50 μm (x20).

Supplementary Figure 3 Neuronal tracing with BDA in JBG-2. The figure shows an absence of associative projections from the dorsal prefrontal cortex (dPFC) to areas of the lateral subthalamic nucleus, STN (dashed lines). The three rows display the anterior, central, and posterior segment, respectively, which is depicted in x1.25 magnification for overview in the left column. Colored miniature windows are the areas seen in detailed x20 magnification in the right column, where no traced fibers are seen in the lateral STN. Only sparse associative projections where found elsewhere in the lateral STN. ZI, zona incerta; cp, cerebral peduncle; Th, thalamus; SNc, substantia nigra pars compacta. Counterstaining with toluidine blue. Scale bars = 1 mm (x1.25) and 50 μm (x20).

Supplementary Figure 4 Neuronal tracing in nucleus accumbens, NAcc, in the upper row, and in septum, Sep, in the lower row. The left overview column is shown in x1.25 magnification marked with a colored miniature window, which is then shown in x20 magnification in the right column. BDA labeled axons are golden-brown and marked with arrowheads. LVa, lateral ventricle anterior part. Counterstaining with toluidine blue. Scale bars = 1mm (x1.25) and 50 μm (x20).

Supplementary Figure 5 Neuronal tracing in the ventral prefrontal cortex, vPFC. The left overview image is shown in x5 magnification marked with a colored miniature window, which is then shown in x20 magnification in the right image. BDA labeled axons are golden-brown and marked with arrowheads. Counterstaining with toluidine blue. Scale bars = 250 μm (x5) and 50 μm (x20).

## Conflict of Interest

The authors declare that the research was conducted in the absence of any commercial or financial relationships that could be construed as a potential conflict of interest.

## Publisher’s Note

All claims expressed in this article are solely those of the authors and do not necessarily represent those of their affiliated organizations, or those of the publisher, the editors and the reviewers. Any product that may be evaluated in this article, or claim that may be made by its manufacturer, is not guaranteed or endorsed by the publisher.
