# Supplementary figures and images for: Anterograde Tracing From the Göttingen Minipig Motor and Prefrontal Cortex Displays a Topographic Subthalamic and Striatal Axonal Termination Pattern Comparable to Previous Findings in Primates
Source: Front Neural Circuits. 2021 Nov 26;15:716145. doi: 10.3389/fncir.2021.716145 (PMC8661455; doi:10.3389/fncir.2021.716145)

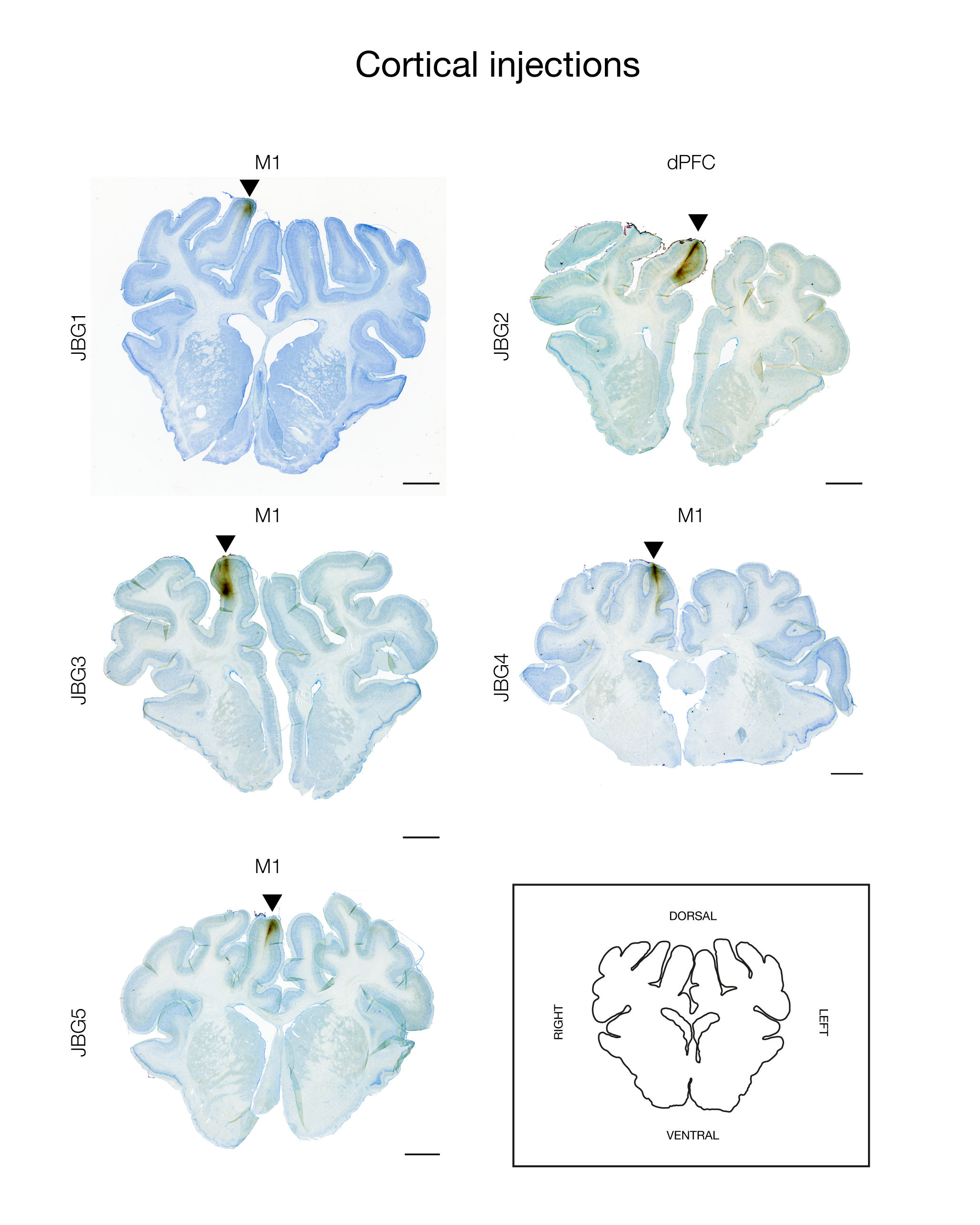

Supplement: Supplementary Figure 1 — Overview of the cortical injection sites in animals JBG1-5. Note the golden-brown BDA labeling of the cortical areas of M1 and dPFC, respectively, marked with arrowheads. Counterstaining with toluidine blue. Scale bars = 5 mm. [file Image_1.TIF]

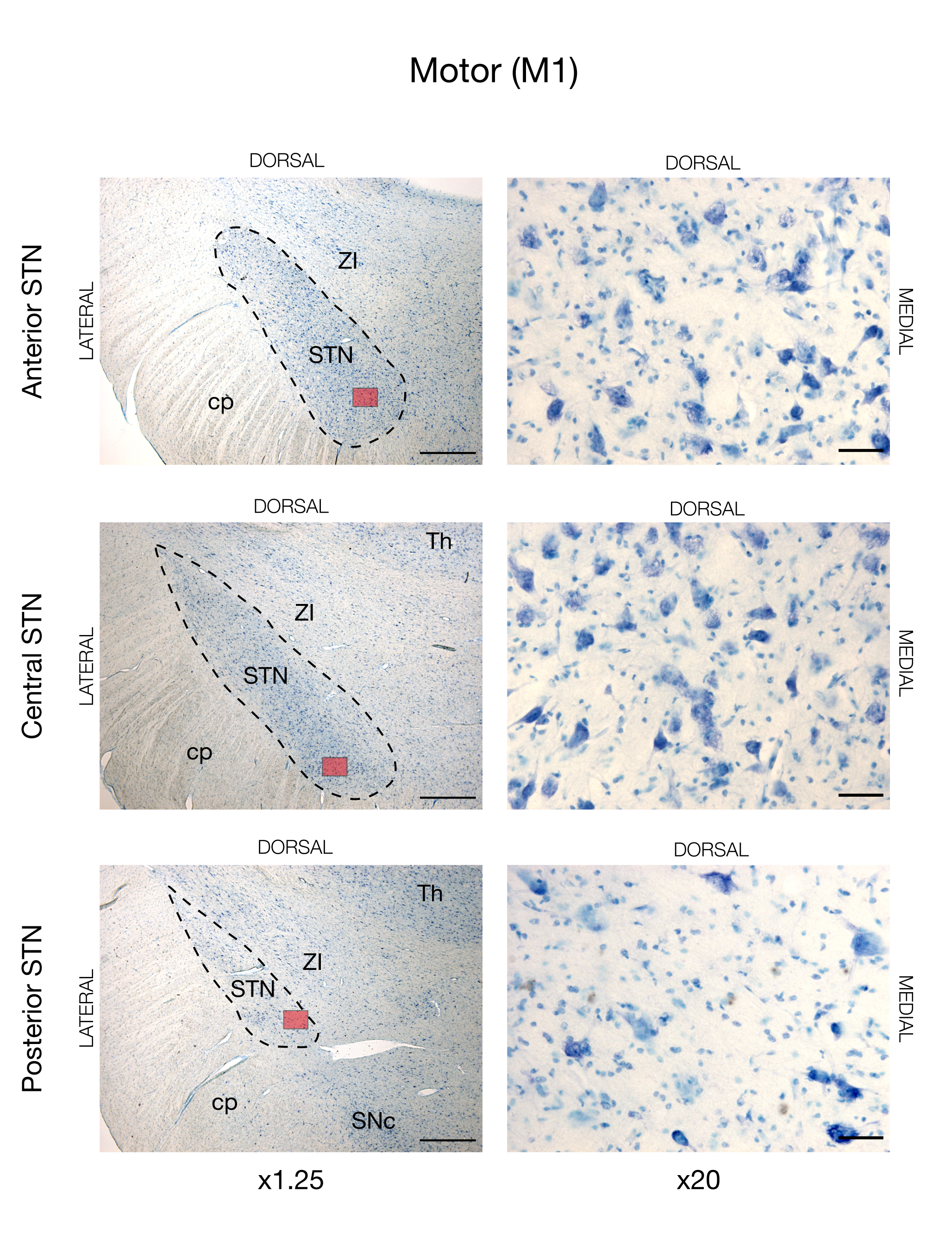

Supplement: Supplementary Figure 2 — Neuronal tracing with BDA in JBG-1. The figure depicts an absence of motor projections from the primary motor cortex (M1) to areas of the medial subthalamic nucleus, STN (dashed lines). The three rows display the anterior, central, and posterior segment, respectively, which is depicted in x1.25 magnification for overview in the left column. Colored miniature windows are the areas seen in detailed x20 magnification in the right column, where no traced fibers are seen in the medial STN. Only sparse motor projections where found elsewhere in the medial STN. ZI, zona incerta; cp, cerebral peduncle; Th, thalamus; SNc, substantia nigra pars compacta. Counterstaining with toluidine blue. Scale bars = 1 mm (x1.25) and 50 μm (x20). [file Image_2.TIF]

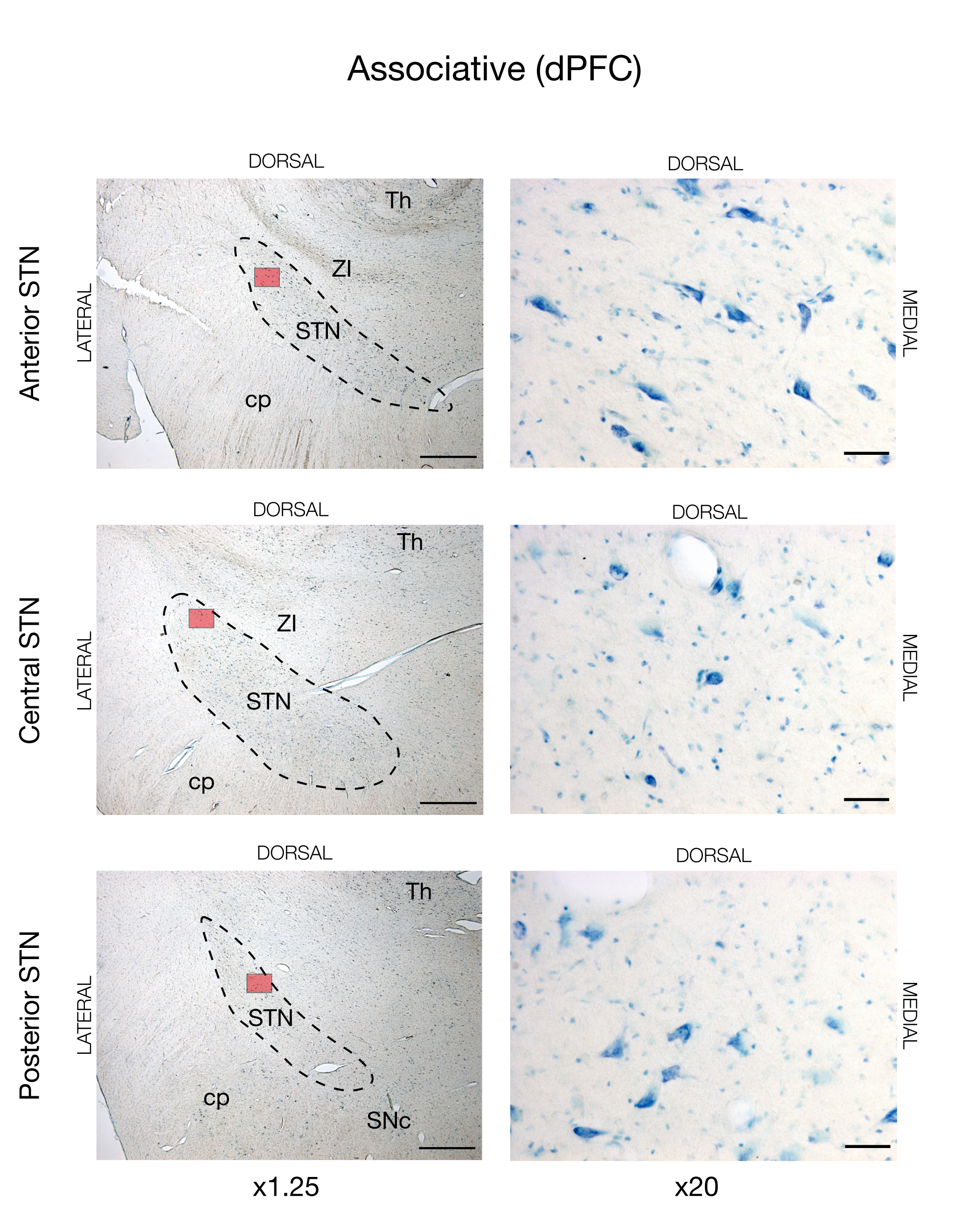

Supplement: Supplementary Figure 3 — Neuronal tracing with BDA in JBG-2. The figure shows an absence of associative projections from the dorsal prefrontal cortex (dPFC) to areas of the lateral subthalamic nucleus, STN (dashed lines). The three rows display the anterior, central, and posterior segment, respectively, which is depicted in x1.25 magnification for overview in the left column. Colored miniature windows are the areas seen in detailed x20 magnification in the right column, where no traced fibers are seen in the lateral STN. Only sparse associative projections where found elsewhere in the lateral STN. ZI, zona incerta; cp, cerebral peduncle; Th, thalamus; SNc, substantia nigra pars compacta. Counterstaining with toluidine blue. Scale bars = 1 mm (x1.25) and 50 μm (x20). [file Image_3.TIF]

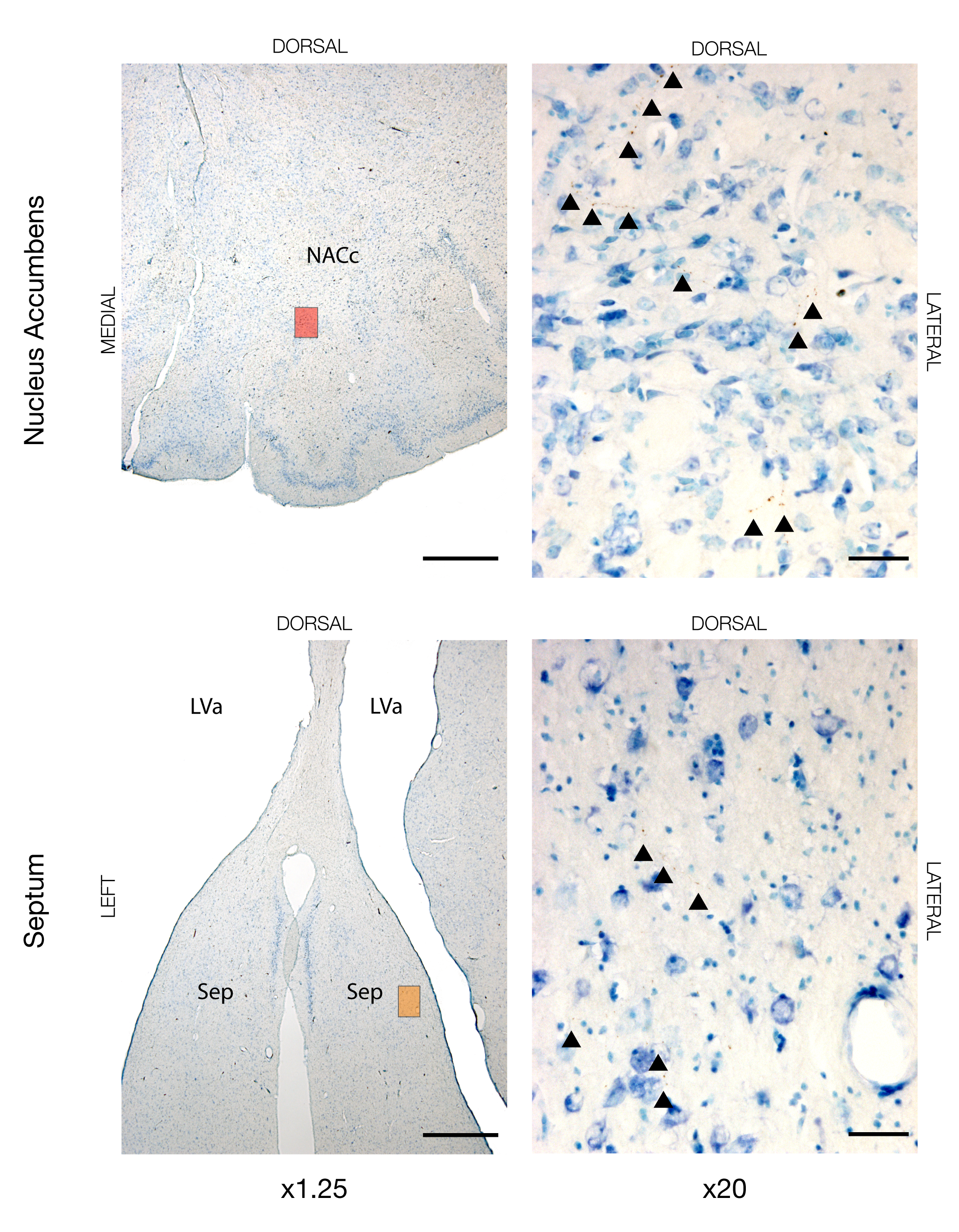

Supplement: Supplementary Figure 4 — Neuronal tracing in nucleus accumbens, NAcc, in the upper row, and in septum, Sep, in the lower row. The left overview column is shown in x1.25 magnification marked with a colored miniature window, which is then shown in x20 magnification in the right column. BDA labeled axons are golden-brown and marked with arrowheads. LVa, lateral ventricle anterior part. Counterstaining with toluidine blue. Scale bars = 1mm (x1.25) and 50 μm (x20). [file Image_4.TIF]

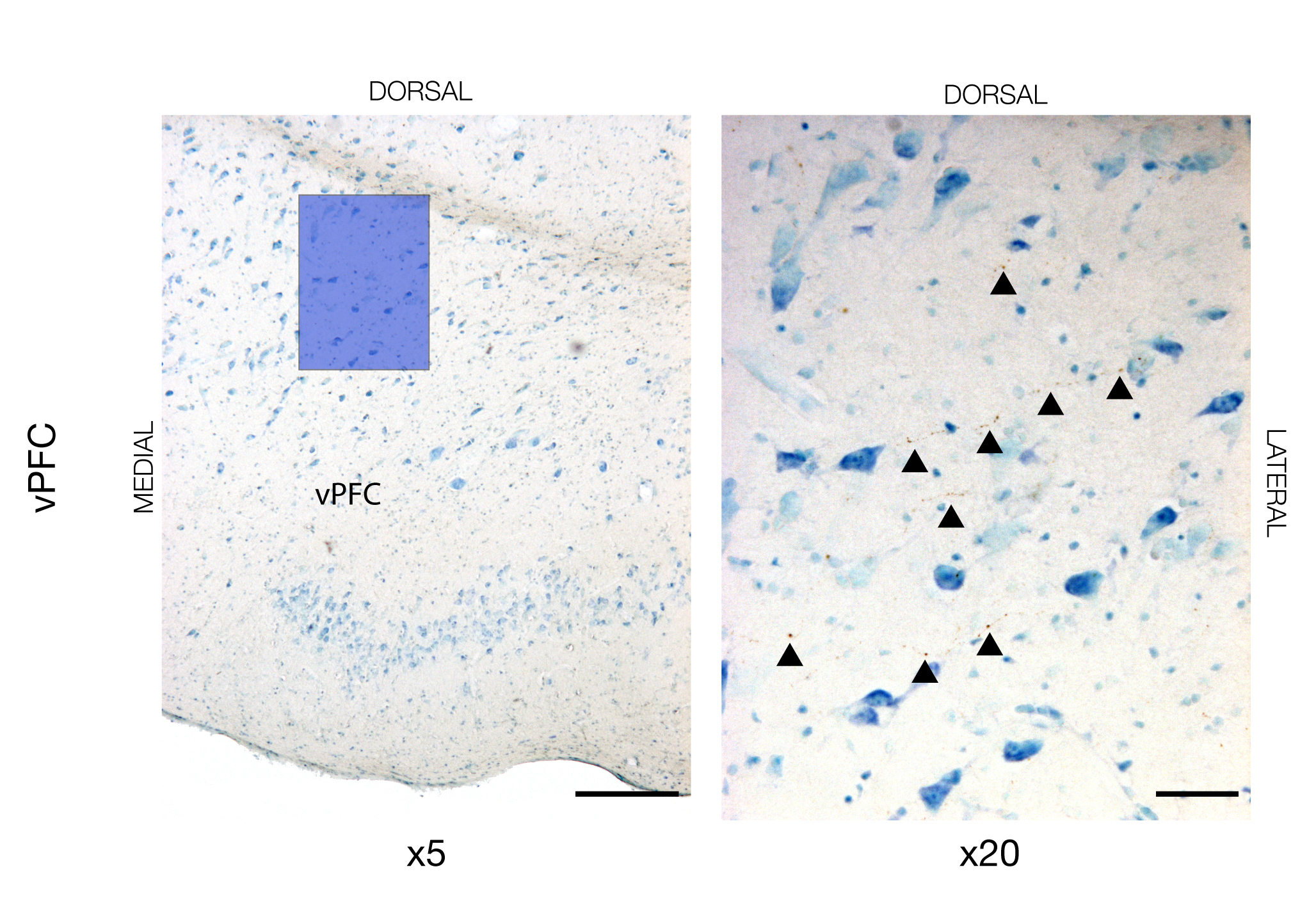

Supplement: Supplementary Figure 5 — Neuronal tracing in the ventral prefrontal cortex, vPFC. The left overview image is shown in x5 magnification marked with a colored miniature window, which is then shown in x20 magnification in the right image. BDA labeled axons are golden-brown and marked with arrowheads. Counterstaining with toluidine blue. Scale bars = 250 μm (x5) and 50 μm (x20). [file Image_5.TIF]
